# Mapping sexual and gender minority inclusion in national adaptation plans globally

**DOI:** 10.1038/s44168-026-00363-5

**Published:** 2026-07-27

**Authors:** Sean Goodwin, Leo Goldsmith

**Affiliations:** 1 Basque Centre for Climate Change (BC3), Edificio Sede 1, 1° Planta, Parque Cientifico UPV/EHU B/Sarriena s/n, Leioa, Spain; 2 Yale School of the Environment, New Haven, CT USA

**Keywords:** Governance, Governance, Interdisciplinary studies

## Abstract

Sexual and gender minorities (SGM) experience disproportionate climate impacts, yet no empirical analysis exists on their inclusion in adaptation planning. We ask: (1) To what extent are SGM included in National Adaptation Plans (NAPs)? and (2) How do legal and social status of SGM differ between countries that include or exclude them? We systematically map all available NAPs (or related documents in their absence, n = 256), analyzing inclusion across transformative capacity and climate justice. Of 198 UNFCCC member states, 23 mention SGM: 65% are SGM-sensitive (acknowledging disproportionate impacts on SGM), 26% SGM-responsive (acting on impacts) and 9% SGM-transformative (addressing systemic drivers of impacts). Higher legal protections (p < 0.05) and positive public opinion (p < 0.01) are significantly correlated with inclusion of SGM in NAPs. Results highlight the need for collaborative, place-based SGM stakeholder engagement to inform adaptation planning, ensuring responses reflect local vulnerabilities and community priorities.

## Introduction

As climate risks escalate, national adaptation planning has become a key policy arena for deciding who gets protected, and who gets left behind. Following COP16 under the United Nations Framework Convention on Climate Change (UNFCCC), parties (especially lower income countries) have been developing National Adaptation Plans (NAPs) to identify medium- to long-term adaptation needs and to formulate and implement strategies to address them^1^. A critical step in formulating NAPs is to identify populations and sectors that are vulnerable to climate change, as this sets the scope of who is included in adaptation goal setting processes and benefits from the distribution of resources and services^2^. Several populations are often defined as being disproportionality impacted by climate change, many owing to social marginalization or exclusion, including (but not limited to) women, children, older adults, and people with disabilities^3^.

Climate justice and intersectionality are key concepts to understanding how social and other inequalities create differential climate impacts based on various facets of individual and community identities. Climate justice highlights how climate change disproportionately impacts marginalized groups, such as those based on nationality, race, or class^4,5^. For example, there is ample evidence that people living in low- and middle-income countries, racialized minorities in high-income countries, women, people who are low-income, people who are unhoused, children, older adults, and people with disabilities are at higher risk for climate-related impacts^6,7^. The emerging focus on how intersectional climate injustices disproportionately impact people with compounding marginalized identities highlights how structural inequality creates varying levels of privilege and disadvantage across societal groups that further shape climate vulnerability^5–7^. Power and marginalization are central to understanding how differential climate vulnerability and intersectionality shape whose voices are heard in decision-making, who has access to resources and services, who may have worsened economic, social, and political outcomes before climate events and who may experience inequitable exposure to climate events. While some facets of identity confer privilege in particular arenas (e.g., being white or male), others contribute to marginalization (e.g., being transgender or gay)^4^. Despite growing awareness of intersectional climate vulnerabilities, sexual orientation, gender identity, gender expression, and sex characteristics remain routinely overlooked (yet essential) in efforts to ensure inclusive climate action.

**Fig. 1:**
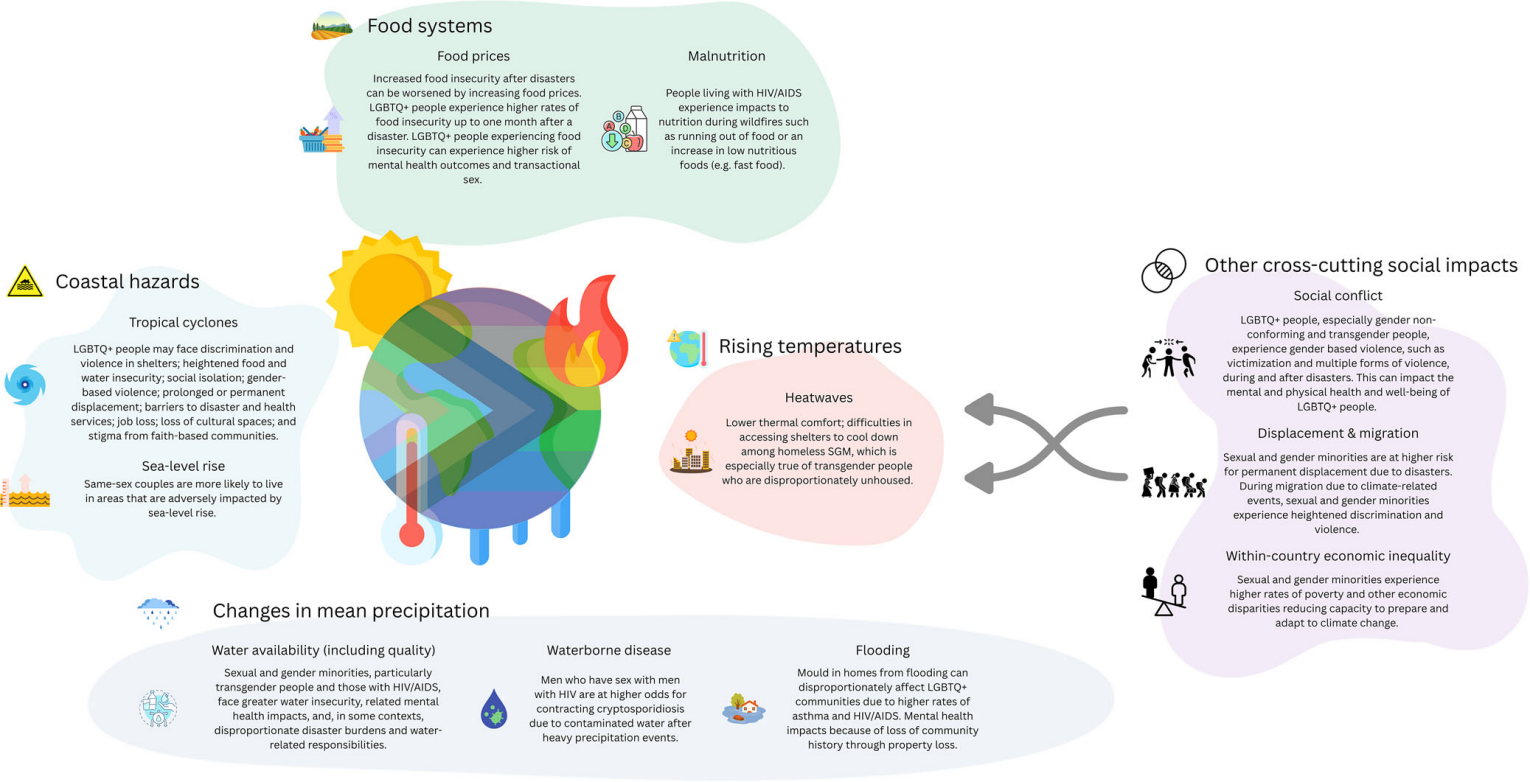
Visual map of existing literature (scientific and gray) documenting specific impacts of key climate risks on SGM populations (based on relevant IPCC risk typology^3^). Includes rising temperatures (heatwaves^102^), changes in mean precipitation (flooding^103,104^, water availability and quality^105,106^, waterborne disease^107^), coastal hazards (tropical cyclones^8,9,69,79,108^, sea-level rise^109^), food systems (food prices^10,11,110^, malnutrition^111,112^), and other cross-cutting impacts (social conflict^70,71,113,114^, displacement and migration^10–12^, within-country economic inequality^8,12^).

Sexual and gender minorities (SGM), encompassing sexual orientation, gender identity, gender expression, and sex characteristics, constitute a critically marginalized group at the intersection of multiple inequalities, yet they remain largely absent from climate justice discourse^4,8,9^. SGM experience social, economic, and health disparities that heighten their vulnerability to climate-related risks, disparities that are exacerbated by continued exclusion from climate planning and policy. For example, SGM can be turned away from necessary resources, temporary emergency shelters, and health care; experience physical, emotional, and sexual violence; and experience disproportionate displacement, water insecurity, unsanitary conditions, and social isolation during climate events (Fig. 1; see Supplementary Table 1 for further details)^8–13^. The intersectional climate justice lens is critical in the context of SGM as various oppressive structures multiplicatively affect them that also increase climate risk exposure.

SGM have also been excluded in key climate governance arenas, for example as participants in climate negotiations, climate mitigation and adaptation planning, and disaster and emergency preparedness, response, and recovery. For example, during the COP29 climate conference in November 2024, the Lima Work Program on Gender, which continues the consideration of gender and advancements in gender equality within the parties, will be extended for ten years. However, despite advocacy from the Women’s and Gender Constituency, a group of women’s civil society and non-governmental organizations (NGOs) that provide input to the UNFCCC processes, to include gender diverse individuals, SGM were not included in the text^14,15^. Despite the evidence of the differential climate impacts that affect them, there is a lack of both theoretical and empirical work exploring what meaningful inclusion means for SGM in adaptation planning processes, and whether it is occurring in practice^16–18^.

The combination of the complex and intersecting climate vulnerabilities experienced by SGM and their lack of inclusion in national adaptation planning processes raises the concern that plans will not translate into actions that support the climate resilience of these populations^19^. NAPs provide a roadmap on what is prioritized, funded, implemented, and evaluated regarding climate adaptation programs. NAPs outline which populations are provided with social protection against discrimination and violence and are included within economic development strategies to build climate resilience. Omission of SGM creates the risk of maladaptive climate action, whereby vulnerability, marginalization and exclusion are worsened rather than improved, an outcome which has been documented in the context of variousmarginalized communities^20,21^. Excluding SGM will ensure that the SGM population will continue to experience disproportionate exposure to climate events, impacts to housing, health, and economic security due to climate events, and lack of resources and funding provided to populations included in NAPs.

This research aims to address two main questions: (1) to what extent are SGM currently included in NAPs? And (2) how does social acceptance and legal support for SGM differ within countries that include or exclude SGM within their NAP? We systematically map the available NAPs for all 198 UNFCCC signatory countries, or where these are unavailable, relevant supporting documents on their development, using a structured search and screening process across official climate policy sources and complementary internet searches (*n* = 256). The systematic mapping of NAPs and other documents was guided by the systematic mapping standards set by the Collaboration on Environmental Evidence^22^ as well as previous work adapting these and similar standards^23,24^ We categorized NAPs or supporting documentation into a three-tier SGM inclusion scale, drawing from feminist literature on the transformative integration of gender in climate adaptation and mitigation applied both in research^25–31^ and practice^16,32,33^. Feminist literature provides a crucial social critique by exposing the structural social, economic, and political systems that create and reproduce climate vulnerability, offering a framework for distinguishing shallow inclusion from gender-transformative implementation of adaptation programs regarding SGM into NAPs. To supplement this, we additionally analyzed how SGM inclusion reflects different aspects of climate vulnerability^3^ and climate justice^34^. To understand the role of social acceptance and legal support in SGM inclusion within NAP, we conducted a statistical analysis of the LGBT Equality Index (Equaldex)^35^ scores of LGBT rights, laws, and public attitudes for each country where available. To our knowledge, this research is the first systematic mapping of SGM inclusion in adaptation planning.

## Results

### There is limited evidence of SGM inclusion

**Fig. 2:**
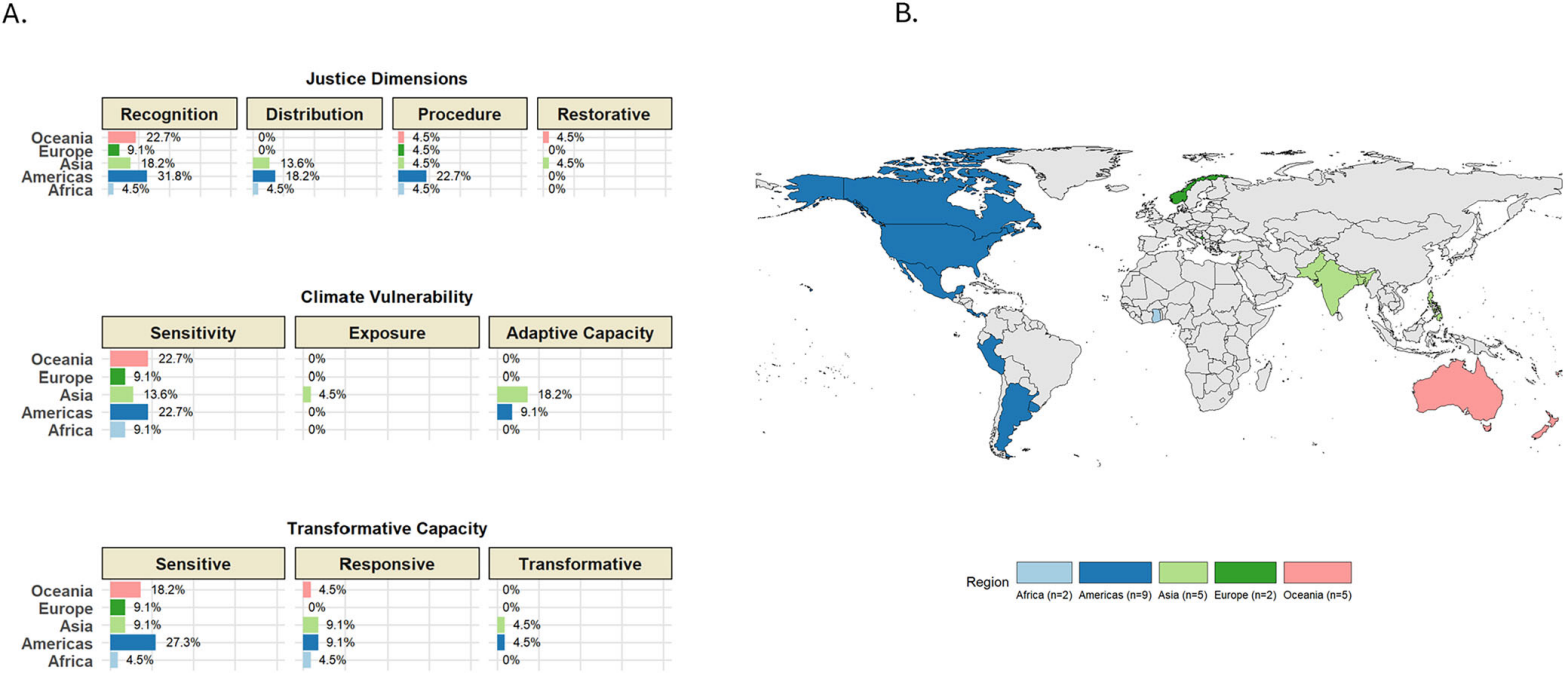
Regional breakdown of key results of analysis of NAPs that included SGM. **a** Regional breakdown of results relating to justice dimensions, climate vulnerability, and the transformative capacity of NAPs that included SGM. **b** World map highlighting countries that included SGM in their nap, coloured by region.

From the 256 NAP or NAP supporting documents retrieved, we found that 23 of the 198 signatories to the UNFCCC, or 12%, included SGM in their NAP or supporting documentation (see Fig. 2, and Supplementary Data 1 for full list of sources retrieved). Of those that did include SGM, 15 mentioned both SGM, while three mentioned sexual minorities and five only mentioned gender minorities. We found that NAPs generally fail to apply an intersectional approach when discussing SGMs or other identities despite growing calls for its inclusion in adaptation^5,8,36–38^. Not all NAPs included women, however, every NAP that included SGM also included women (though rarely clarified whether this referred to a binary understanding of gender or not). Inclusion of SGM usually occurred right after discussions or mention of women. In addition, intersex individuals are rarely included among different SGM, who nonetheless often experience significant disparities in the context of climate adaptation^39,40^.

There was no consistent terminology used across NAPs or related documents to describe SGM, and few specified which groups were considered beyond broad collective labels. Terms included various acronyms, such as LGBT (lesbian, gay, bisexual, transgender), LGBTQ (adding queer), LGBTQI (adding intersex), LGBTQIA (adding asexual), and LGBTQIA+ (acknowledging identities beyond those listed). Some documents also used 2SLGBTI+ to recognize Two-Spirit identities in North America. Other terms included ‘transgender’’, ‘gender diverse’’, ‘gender minorities’’, ‘sexual orientation’’, and ‘gender identity’’.

### Limited evidence of inclusion of transformative capacity in NAPs

We analyzed the degree to which countries included SGM in their adaptation planning processes on a three-tier scale of their capacity to produce transformative change (Fig. 2A, bottom panel). This framework was adapted from leading literature evaluating the integration of gender applied both in research^25–31^ and practice^16,32,33^. We categorized NAPs into one of these levels based on the highest possible level we could characterize them as reaching given the available information.

Our results demonstrate how most NAPs reflect superficial inclusion of SGM. We labeled most NAPs (or supporting documents) as *SGM-sensitive* (65% of all countries that included SGM in their NAP or supporting documentation, hereafter simply referred to by their percent value of each area of analysis), as they acknowledge SGM-related risks but lack concrete actions. This pattern is especially evident in the Americas and Oceania. For example, Canada’s NAP identifies 2SLGBTQ+ people as a marginalized group but does not propose specific measures^41^. *SGM-responsive* plans outline specific actions for SGM, such as India’s inclusion of transgender women in microcredit programs, but lack documented integration into broader policy areas or monitoring (26%)^42^. *SGM-responsive* NAPs were most common in the Americas and Asia. *SGM-transformative* NAPs (such as those from Argentina^43^ and Bangladesh^44^) go beyond recognizing the vulnerability of SGM to climate change (9%). These plans evidenced transformative capacity in how they detail ways of embedding structural changes by integrating SGM into decision-making processes, establishing concrete actions and measurable indicators for inclusion and intention to implement climate adaptation programs that invest in social infrastructure and social protections. This marks an advancement beyond sensitivity or responsiveness by moving past acknowledgement or isolated actions to specify changes that confront the diverse social, economic, and political structures that produce SGM vulnerability to climate change. Given the nascent state of research and practice, this is articulated in terms of transformative *capacity*, as actual transformative outcomes are difficult to evidence from planning documents alone (see Methods, Table 1 for further detail on the coding process). For instance, Argentina’s NAP explicitly sets out mechanisms to evaluate how LGBTI+ people are incorporated into planning across several policy domains (e.g., sovereignty, habitability, and care). Bangladesh ensures gender-diverse individuals participate in community-based adaptation and climate financing, detailing outreach strategies and a raft of actions to be taken. Though it is unclear, we hypothesize that lower levels of inclusion of SGM in NAPs may be due to the relatively recent emergence of the topic at an international level and the ongoing politicization of SGM identities^45^ (see Supplementary Table 2 for a more detailed analysis of each country’s inclusion).

**Table 1:** Clarification for how SGM-sensitive, responsive and transformative were coded

Coding Concept	Definition in relation to SGM inclusion in NAPs	Example inclusion criteria
SGM-sensitive	Where a NAP mentions SGM in some capacity, providing a descriptive account of SGM vulnerability to climate change.	A NAP includes a paragraph describing how SGM individuals experience disproportionate heatwave impacts because of exclusion from formal support services, without detailing any actions to address these vulnerabilities.
SGM-responsive	A NAP that satisfies the definition of SGM-sensitive, while also elaborating actions taken to address SGM vulnerability to climate change.	A NAP notes barriers SGM individuals face in accessing evacuation centers and commits to developing non-discrimination guidelines for shelters, with a linked indicator measuring the rollout and compliance of these guidelines across provinces.
SGM-transformative	A NAP demonstrates SGM-transformative capacity when it moves beyond acknowledging SGM vulnerability or proposing isolated actions, and instead identifies the specific social, economic, or political drivers of that vulnerability and outlines actions explicitly designed to address them. These actions are linked to concrete indicators within the Monitoring, Evaluation, and Learning framework.	A NAP demonstrates SGM-transformative capacity when, for example, Argentina’s Gender and Diversity cross-cutting approach identifies discriminatory barriers faced by LGBTI+ populations in accessing safe housing, health services, and employment (as referenced in the “sovereignty,” “habitability,” and “care” axes) and links these to broader social and institutional drivers of vulnerability as well as specific indicators to be measured over time.

It is important to note that inclusion or transformative capacity on paper does not necessarily translate into inclusive or transformative outcomes in practice. The inclusion of SGM in NAPs is often influenced by political realities that can hinder progress. In Argentina, current political shifts under President Milei, such as the ban on gender-affirming care (under Decree 62/2025, DNU-2025-62-APN-PTE, Provisions. Buenos Aires, 06/02/2025.), question the future of the transformative potential of the country’s NAP for SGM (especially trans people). Similarly, in the United States, the rollback of diversity, equity, and inclusion efforts under the Trump administration demonstrates how SGM inclusion can be swiftly dismantled^46^. Meanwhile, Thailand’s initial inclusion of SGM in planning documents showed transformative capacity, but these commitments were not reflected in the final published NAP, highlighting a disconnect between inclusion on paper and actual implementation. Thus, our results should be interpreted with the understanding that inclusion in NAPs or supporting documents does not necessarily translate to inclusive action. However, both serve as useful proxies for identifying national priorities on this issue. While commonly used in scholarship, reducing inclusion to a three-tier scale inevitably simplifies the diversity of inclusion captured by our results. However, this limitation has a minimal impact in the context of our study, given the overall lack of detailed information due to limited inclusion. As such, a more streamlined analytical framework is justified.

### A lack of focus on the root cause of vulnerability

We analyzed how NAPs that included SGMs claimed to address their climate vulnerability according to the definition of the Intergovernmental Panel on Climate Change (IPCC). This definition highlights how vulnerability is a function of a population’s sensitivity and exposure to climate hazards, adjusted to account for their capacity to adapt^3^. Sensitivity, the most addressed form of vulnerability (74%), refers to the acknowledgment of the increase level at which SGM are impacted by climate impacts. For instance, Canada’s National Adaptation Strategy highlights the increased severity of climate impacts on SGMs, particularly due to their exclusion from social determinants of health^41^. Adaptive capacity then focuses on enhancing the ability to cope with these impacts (26%). Mexico’s NDC, for example, includes resources aimed at improving institutional capacity with applying a gender and human rights perspective to adaptation^47,48^.

Exposure, the least addressed form of vulnerability (4%), refers to the systemic factors that create differential impacts. Exposure is not only a function of biophysical hazard but is also shaped by structural inequality, urban planning decisions, and socio-political histories (e.g., housing or zoning laws that place certain population in at-risk areas that do not have access to basic infrastructure)^49,50^. Addressing exposure can therefore offer a more fundamental entry point to reducing vulnerability, by preventing certain groups from being placed at risk in the first place. Nonetheless, sensitivity and adaptive capacity remain critical, as it is prudent to plan for some level of exposure by strengthening the systems that shape how impacts are experienced and managed^51^. Bangladesh’s NAP addresses this by attempting to increase coverage of access to water, hygiene and sanitation facilities to gender-diverse populations^44^. Sensitivity was most discussed in the Americas and Oceania, while adaptive capacity and exposure were more prevalent in NAPs from Asia.

### Minority of NAPs move beyond recognition justice

Climate justice can be understood across several dimensions^34^, which appeared with varying frequency in our analysis. Recognition (86%) was the most common, with many plans acknowledging the disproportionate impacts of climate change on SGMs. Procedural justice (39%) was less frequent, exemplified by Mexico’s NAP, which calls for SGM participation in adaptation policy design. Distributional justice (35%) appeared in a smaller number of cases, such as Canada’s NAP, which highlights the need to consider diverse health determinants for 2SLGBTQ+ populations. Restorative or reparational justice (9%) was the least observed, though Fiji’s NAP offers a notable example by reframing SGMs as “active agents of change” rather than solely “vulnerable groups,” in recognition of historical exclusion. However, consideration of multiple dimensions of climate justice could be more often seen in the Americas, except for restorative justice, that was more often considered in NAPs for Asia and Oceania.

### Public opinions and legal protections support inclusive adaptation planning for SGM

Some countries with weak legal protections and negative public perceptions of SGM—such as Pakistan, Lebanon, and Bangladesh—included gender minorities in their climate plans, possibly due to domestic advocacy (e.g., Pakistan’s 2017 Census and UNDP framework)^52^. In contrast, countries like Iceland, Spain, and Germany, despite strong legal protections and positive public opinion, did not, which may reflect limited data or awareness; similarly, Australia omitted SGM from national plans despite commissioning research on their climate vulnerability in other regions (e.g., Fiji)^53^. These unexpected patterns highlight the need for more research to understand the drivers of inclusion and exclusion of SGM in climate policy.

**Fig. 3:**
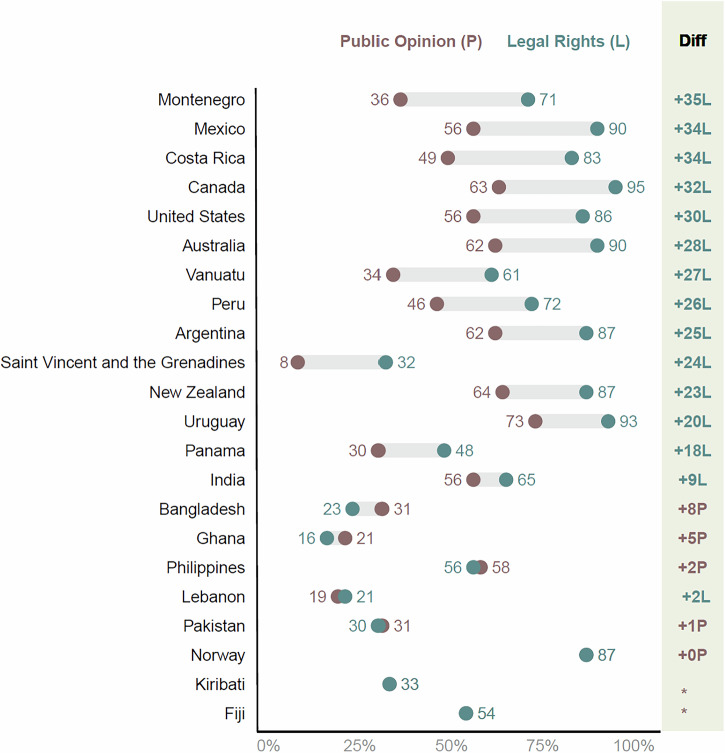
Graph summarizing Public Opinion and Legal Rights scores (Equaldex) for countries that included SGM in their NAP. The figure plots Public Opinion (brown dots; global mean = 34.5, global median = 32.5) and Legal Rights (green dots; global mean = 48.1, global median = 49) on the x-axis, with country names on the y-axis. The difference between these two scores is displayed in the right-hand panel.

To explore potential relationships, we drew on the Equaldex, a robust crowdsourced database of global SGM legal and public-opinion metrics, also used in indices like UC Berkeley’s Inclusiveness Index. Based on a Wilcoxon Rank Sum test using this data (where available, *n* = 171), we find that countries that included SGM in their NAPs or supporting documentation tend to have higher public opinion (*p* = 0.0014, rg = -0.430) and stronger legal protections (*p* = 0.0141, rg = -0.331) for SGMs (see Supplementary Table 3 for details). While both factors appear related to inclusion, the relationship with public perception is stronger, and the wide diversity of scores among countries that did include SGM highlights the complexity of these dynamics. However, among the 23 countries, on average, legal rights were much higher than public perception scores and higher than the global mean (48.1, Fig. 3).

This analysis was used to understand inclusion through the lens of how SGM are treated across countries at a higher level, though we acknowledge that future research ought to explore other determinants of inclusion^54,55^. Further research is required to investigate the factors that shape SGM inclusion in climate policies and adaptation planning across scales (from local to national) to understand why some plans include SGM while others do not. It is notable, for example, that the extent and depth of inclusion do not follow a clear pattern between countries of the Global North and Global South. Both NAPs coded for transformative capacity come from the Global South (Argentina and Bangladesh), indicating that level of SGM inclusion cannot be attributed solely to economic factors. This should include more granular analysis of which sub-populations (e.g., sexual versus gender minorities) are named in adaptation efforts, to assess whether inclusion is genuinely intersectional or limited to broad umbrella terms (e.g., LGBT). It is important to note that while indices like Equaldex offer useful comparisons, they rely on diverse sources (e.g., national surveys) that may privilege certain SGMs over others, such as the frequent exclusion of trans people.

## Discussion

Building on existing knowledge, our analysis not only incorporates a broader range of documentation to map SGM inclusion in developing NAPs, but also advances the field in two additional ways: (1) consulting a wider range of documentation on the development of national adaptation priorities, and (2) by investigating the quality of SGM inclusion in NAPs in the context of how they support transformative capacity for SGM, and how they claim to reduce their vulnerability and support climate justice through the process. We found 23 NAPs to include SGM which is a much larger amount compared to what others have found. The NAP Global Network, found 11% of published NAPs (n = 6, including only those officially submitted to the UNFCCC) included reference to LGBTQ+ populations^56^, while others have found only three signatories reference SGM in Nationally Determined Contributions^57^. It is unknown what terms were screened to identify inclusion or if systematic methods were used within this literature. The limited and uneven way in which gender (whether including SGM or not) is incorporated into adaptation practice more generally has further been noted by previous scientific studies^25,28,58^.

Our findings suggest that favorable public opinion toward SGMs often precedes legal protections, underscoring the role of advocacy and liberation movements as key levers for advancing SGM inclusion in NAP processes^59^. There are opportunities to learn from practices of countries with favorable public perceptions of SGM, such as Cabo Verde that did include SGM in their NAP even though their legal protections of SGM lag behind social acceptance. Cabo Verde has long been acknowledged as a beacon of acceptance for SGM in the African region^60^, anecdotally linked to its strong gender equality movement^61^. Our results nonetheless highlight the key role of legal protections for SGM inclusion. These results should be interpreted with the understanding that they focus solely on national-level policy processes, which, while offering valuable initial insights, may overlook regional or local efforts. It is important to note that the last few UNFCCC COPs (e.g., Egypt and Saudi Arabia) have been in countries not only with low legal protections for SGM but further actively criminalize and persecute them in spite of ongoing advocacy against these host locations^62–64^, which may further hamper progress on SGM inclusion.

Our findings further contribute to earlier work that identifies a connection between support for SGM in climate adaptation and the broader gender equality movement. Our analysis shows that when NAPs mention SGM, they almost always include women, often positioning SGM language directly after discussions on women. This reflects previous work that highlights shared vulnerabilities between women and SGM^13^ and shared history of rights-based activism^65–67^, especially in the context of feminist political ecology^68^ and disaster-risk reduction^69^. An intersectional approach can open conversations where SGM can also include women, and that these compounding marginalized identities can experience multiplicative impacts of climate change. There is an emerging area of literature that highlights how both women and SGM are disproportionately affected by gender-based violence during climate disasters and conflict^13,70–72^. Emphasizing the interconnections between these movements highlights why a power-aware intersectional approach is essential in climate negotiations on gender and adaptation. Efforts focused on a single identity (e.g., cisgender women over transgender women) overlooks synergies possible to uplift multiple groups because of shared underlying structures of oppression, such as misogyny and other systemic inequities^73,74^. Moving beyond a surface-level, binary understanding of gender and sex would be a significant step toward addressing these power imbalances.

While progress is still needed, efforts to include women in NAPs have already driven transformative adaptation by addressing gender-specific needs, increasing participation, and directing targeted funding, particularly in the Global South^25^. In analyzing differences in inclusion between the Global North and Global South, it is important to recognize that countries in the Global South bear a disproportionate burden from climate change and, consequently, the need to plan for adaptation^75–78^. By extension, SGM communities in these countries face an additional disproportionate risk of maladaptation when adaptation planning insufficiently addresses their needs, as observed in some NAPs from Global South countries that were only partially SGM-sensitive or responsive. It is possible that even if a NAP does not include SGM, a more formalized NAP and a country with more resources for climate adaptation program implementation could have a beneficial impact on SGM. However, there are many cases in which countries that have both have SGM populations who experience discrimination, violence, and disproportionate impacts of climate change, such as the United States, Australia, and Japan^8,79–82^. This suggests that inclusion of SGM in the NAP is, at least, a necessary first step in addressing those harms.

Our results suggest that learning from political movements could strengthen the inclusion of SGM in adaptation planning, and that broadening current gender-sensitive approaches to explicitly embrace gender diversity could further advance SGM visibility and inclusion. Work by UN bodies and the NAP Global Network signals growing institutional support for SGM inclusion in adaptation planning. For example, the NAP Global Network’s 2019 supplement to the UNFCCC Technical Guidelines highlights the importance of including sexual orientation and gender identity in NAPs^83^, and the Global Goal on Adaptation calls for “intersectional approaches” in adaptation action^84^. While our results do not confirm a direct link between such guidance and inclusive NAPs, they underscore the need for further research into how institutional frameworks like these can empower SGM inclusion.

In addition to signatories and the UNFCCC secretariat, civil society has played a critical role in further advancing SGM inclusion across adaptation policies. In previous COPs, there have been some, though limited, events with a focus on SGM and intersectionality from countries, such as Canada and non-profits, such as OUT for Sustainability (OUT4S) and SustainUS^85,86^. The Women Environment and Development Organization (WEDO), as the focal point of the Women’s and Gender Constituency (WGC), has been an advocate of both women and SGM inclusion in the UNFCCC processes^85^. Likewise, the Gender and Environmental Data Alliance (GEDA)^87^ and the Sexual and Reproductive Health Right (SRHR) & Climate Justice Coalition^88^ have made important strides to increase awareness of this topic. However, these advancements follow an observed trend of exclusion of SGM advocacy groups in various intergovernmental processes relating to governance mechanisms for addressing the climate crisis^59^.

Several practical policy recommendations can be made at the national and international level to bolster the position of SGM in climate adaptation planning. At the national level, these include^89^:

1. Including and ensuring direct participation of SGM and recognized representative organizations in adaptation planning.
2. Collecting disaggregated, intersectional sexual orientation and gender identity data to us as indicators in vulnerability assessments, such as those proposed by the World Bank.
3. Targeting and engaging SGM communities in educational and risk awareness campaigns on climate impacts.
4. Working with recognized organizations and community centers to develop adaptation-specific capacity.
5. Establishing grants prioritizing SGM and other marginalized groups, incorporating intersectional criteria and aligning with international equity standards and feminist climate adaptation literature on transformative change.
6. Promoting coordinated national and international action to integrate SGM considerations into environmental policy and multilateral climate processes, leveraging partnerships, mandates, and inclusive indicators.

At the international level, and in preparation for future COPs, our findings highlight the need to explicitly include SGM and intersectionality in gender and climate policy discussions, particularly regarding adaptation planning, climate finance, and loss and damage. For example, institutional efforts like the Lima Work Program on Gender and the Women’s and Gender Constituency could extend outreach to SGM groups, include them in decision texts, and codify their representation. We suggest that strengthening institutional support for the intersectional inclusion of SGMs could advance the Global Goal on Adaptation by reinforcing commitments to gender-responsive, inclusive NAPs and enabling the use of equity-focused indicators to track adaptation progress. We further argue that COP negotiations, which has a strong impact on how marginalized communities are considered in adaptation planning, should only be hosted by governments that uphold basic human rights, including protections for SGM and their ability to participate without fear of violence or persecution.

There are further several areas of current practice that span the local to global level that provide inspiring ways forward in this area. Although there is still limited evidence directly linking the meaningful inclusion of SGM communities in adaptation planning to measurable improvements in adaptation outcomes, an emerging body of work points to the significant opportunities such inclusion can create for both communities and adaptation practice more broadly. For example, the Waria Crisis Center in Indonesia (an organization supporting trans communities in climate adaptation and disaster risk reduction) illustrates how dedicated spaces can act as critical enabling conditions. Such spaces help surface and strengthen the inherent resilience within SGM communities, particularly through social capital, and demonstrate how this resilience can be mobilized in the face of evolving climate risks^90^. Additional studies underscore that organizations of this kind can serve as effective partners for national and subnational governments in consultation processes when they receive adequate financial support and formal recognition of their expertise^91^. The number of local, community-based organizations and international NGOs serving SGM populations and engaging with climate resilience is also growing, such as The 519 in Toronto, Canada and the Parliamentarians for Global Action, offering promising entry points for SGM participation in adaptation planning practice across scales and reduction of discrimination and violence that can arise due to climate events^92,93^.

To conclude, from a systematic mapping of the inclusion of SGM in NAPs and supporting documents globally, we found seldom reference to the special risks faced by SGM. Further, even among those countries that did recognize SGM risks, inclusion was limited, did not include an intersectional approach, and excluded SGM subpopulations. While we welcome the progress made to date, further work needs to be done to more comprehensively include SGM in NAP processes. Future action must better support exposure reduction and adaptive capacity, as well as ensuring adaptation benefits are distributed among the various communities that encompass SGM. Our statistical results suggest that efforts towards improving social perception of SGM could be a key lever towards improving inclusion within NAPs, followed by better legal protections of SGM. However, changing public perceptions and legal protections of SGM cannot be done exogenously. Our recommendations have thus been targeted towards intergovernmental processes (like the UNFCCC and IPCC processes) that have the power to shape national priorities on climate change adaptation planning. Within these processes, the unique challenges faced by SGM demand not just acknowledgment, but visibility, inclusion, and strident action to prevent their continued erasure.

## Methods

Between November 2023 and November 2024, we systematically mapped NAPs or, in the absence of an official NAP, documentation related to national adaptation planning, for all 198 countries that are members of the UNFCCC. This was guided by the systematic mapping standards set by the Collaboration on Environmental Evidence^22^ as well as previous work adapting these and similar standards^23,24^. This process can be summarized as (1) setting the objectives and scope of the mapping, (2) establishing a search and screening process, and (3) coding and extracting relevant information. Throughout these steps, an equal workload was assigned to each author (i.e., the number of countries to search, screen, and code information for). These steps are outlined below.

### Objectives and scope of the mapping process

A mapping protocol was first established between the authors to guide what kind of information would be collected. The protocol framed what kind of subject, intervention, and outcome (or SIO) guided the search process. In terms of the subject, our study focused on SGMs. In the context of our study, SGMs include but are not limited to individuals who identify as lesbian, gay, bisexual, asexual, transgender, non-binary, Two-Spirit, queer, and/or intersex. This choice of language was made based on terminology used in previous work with a similar focus in different fields^13^. Common language describing these communities was a challenge to the screening procedure, our approach to which is outlined below. The intervention then related to the documentation of adaptation planning processes on the national level. The outcome we searched for was the inclusion of SGMs within national-level adaptation planning processes, especially in relation to climate vulnerability. Following the IPCC, we understood vulnerability to mean planning for reducing exposure or sensitivity, or otherwise supporting adaptive capacity, of SGMs^3^.

### Search procedure

The search procedure describes the process of how information about national adaptation planning processes was systematically retrieved. Given that there is diverging progress regionally on national adaptation planning globally, a variety of sources had to be consulted through a layered approach. This means that we did not limit ourselves to only countries with final plans. We also included relevant and current information about preparatory work being undertaken to prepare NAPs to gain insight into how planning processes incorporate the needs of SGM.

First, the UNFCCC NAP Central database was consulted both for published NAPs from “developing”^94^ and “developed”^95^ countries. For countries without an NAP, other databases were consulted. In the case of Europe, a regional database (ClimateADAPT^96^) exists that tracks progress for countries in the region. The most recent adaptation plans or strategies were obtained in this way.

**Fig. 4:**
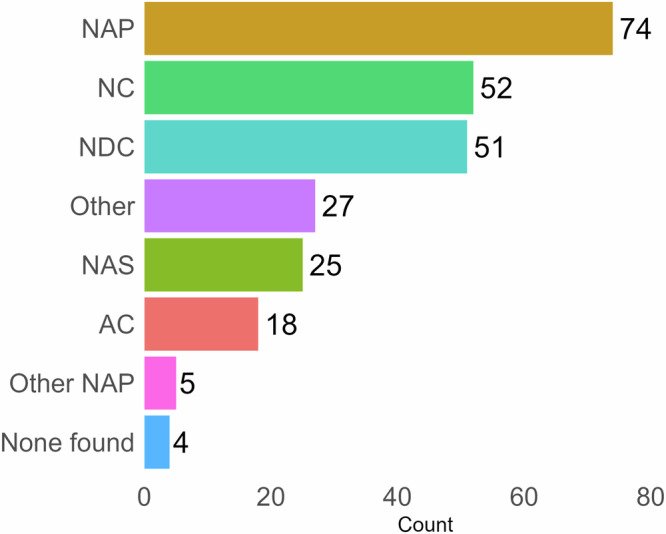
Summary of documents retrieved and analyzed by Type (y-axis): “*NAP*” (National Adaptation Plan, published on the UNFCCC or government websites), “*NC*” (National Communications submitted to the UNFCCC containing information about progress towards national adaptation planning), “*NDC*” (Nationally Determined Contributions submitted to UNFCCC that contain information about national adaptation planning progress), “*Other*” (Preparatory or other documentation describing the process and progress towards developing a NAP on government or organization websites supporting this process, for example GCF Readiness Proposals), “*NAS*” (published National Adaptation Strategies, usually published on government websites), “*AC*” (Adaptation Communications submitted to the UNFCCC containing information on national adaptation planning progress), “*Other NAP*” (NAPs published on government or other websites not found on the UNFCCC website), and “*None found*” (where no NAP or other documentation could be found, including Holy See, Iran, North Korea, and Libya).

For remaining countries that did not appear in any of these sources, an Internet search was conducted in English using Google to identify any additional NAPs, or in their absence, documentation relating to their development. To keep results concise, we used the search terms of the name of the country in addition to “national adaptation plan” or “national adaptation strategy”. The first ten results were reviewed to identify reliable and current information. Reputable sources in this regard included official governmental websites or those of NGOs or similar entities acting on behalf of countries to support them in adaptation planning processes. The only sources retained in our final analysis included those from official government websites, the Green Climate Fund (Readiness Proposals prepared for NAP development), as well as reports from other United Nations bodies (e.g., UNFCCC, FAO),

To cross-reference these results, or complement them if nothing was otherwise found (especially because Internet searches were only conducted in English), additional documentation was searched for in both the UNFCCC nationally determined contributions and adaptation communications databases, which has previously been used in studies to understand adaptation planning priorities^97^. As a final step, the Climate Change Laws of the World database was consulted to identify any documentation relating to adaptation planning processes at the national level. Where an official NAP did not exist, all documentation found through these additional searching steps was cataloged for the combined insight they provide into national adaptation planning processes and priorities (Fig. 4 below). These additional steps were also important to mitigate the bias introduced by only conducting initial internet searches in English, as documents retrieved through additional searches were often in local languages. The results of the search process are summarized in Fig. 4 below.

Our focus on national-level plans and documentation inherently excludes progress on SGM inclusion in adaptation planning at sectoral, regional, or local scales (e.g., city-level efforts), which may demonstrate significant advancements compared to the national scale. We prioritized national-level analysis due to the availability of relatively uniform documentation facilitated by international collaboration under the UNFCCC process. While this approach was pragmatic given the complexities of harmonizing global efforts across scales and sectors—particularly due to differences in language and framing—it inevitably overlooks valuable progress at other levels. Future research should address these gaps, though it will require considerable resources to overcome the challenges of accessing and standardizing diverse data from multiple scales and sectors.

### Screening procedure

The screening process relates to reviewing all results of the search process (n = 256 documents in our case) to whether they meet the inclusion criteria set by the study relating to the SIO. Note that the total number of documents exceeds the number of UNFCCC signatories (n = 198) as all documents retrieved through searches were retained and analyzed. Screening the documentation was challenging in the context of our SOI given the great diversity of terms used to describe SGM. To overcome this, we searched all plans for explicit reference to several words that cover different facets of the population:

- Gender
- Sex
- Vulnerable
- Gay
- Lesbian
- Bisexual
- Orientation
- Identity
- Transgender
- Non-binary
- Two-spirit
- Common abbreviations relating to these identities, including SOGI, LGBT, and LBTI.

The first three search terms allowed us to understand all references to gender, sexuality, and how the specific vulnerability of any group (whether SGM or not) are included, which we found to be a robust way of identifying whether the documents included our subject. Some search words were duplicated (i.e., gender within transgender), though we nonetheless added them as a redundancy measure in the search procedure to improve reliability. For the same reason, stems of words were also used where relevant (e.g., “vulnerab” to capture all iterations of “vulnerability”). This resulted in the inclusion of documentation from 23 countries. Where documentation was not in English, they were machine translated using Google translate, and were read in their entirety to try to mitigate the risk of translation error, rather than relying on common search terms alone. This usually meant looking more closely at sections discussing vulnerability and gender, which are common themes within the documents retrieved.

### Coding and data extraction

We analyzed the documentation relating to the 23 countries who provided some level of inclusion of SGM in NAPs following the content analysis approach described by Schreir^98^. Analysis was split equally between both authors, and the results were discussed and compared to ensure agreement with the codes applied. Although the primary analysis was divided between the two authors, both were able to review and analyze the entire sample due to its relatively small size. The brevity of the information within each document further facilitated detailed cross-checking and allowed the authors to reach consensus on the coded material. The authors met several times over the course of coding and data extraction to discuss any discrepancies that occurred.

We used three categories of codes that were applied in a deductive/theory driven fashion according to their definition in existing literature. This includes (1) codes on what kind of climate vulnerability is addressed in relation to SGM within the documentation following the IPCC definition (i.e., reducing exposure, reducing sensitivity, or supporting adaptive capacity), (2) what kind (if any) of social justice dimensions are supported in relation to SGMs (understood as procedural, recognition, distributive, and restorative^34^), and (3) whether the inclusion of SGMs in climate adaptation planning better fits the definition of being “*SGM-sensitive*”, “*SGM-responsive*”, or “*SGM-transformative*” following pertinent literature in this field from research^25–31^ and practice^16,32,33^. Coding and synthesizing the data focused on finding references to these three categories within the set of documents found to be in-scope of the study, following previous similar stock-taking approaches^23,24^. Because the language of SGM-sensitive, responsive, and transformative lack widely accepted definitions, additional information is provided in the table below.

In terms of the final category relating to transformation, it is important that “transformation” is defined in numerous ways even within the context of climate change adaptation alone^99^. Our adaptation of this theory to the goals of our study was because of its roots in feminist ideology in relation to understanding the impact of different gender-based interventions particularly in the context of climate change. It was useful in our context because of its previous applicability to the subject matter we were analyzing (adaptation planning) particularly towards delineating planning instruments with more modest impacts (i.e., being SGM-sensitive), more robust impacts (i.e., SGM-transformative), as well as being able to identify those planning processes that occupy the space in between these levels (i.e., SGM-responsive). The definition of transformation we have employed therefore relates to more recent literature that highlight several key features of the concept in action, for example, its departure not only from “business as usual” or addressing underlying drivers of the undesirable change or outcomes that are the focus of an area of policy, but rather move beyond this by encouraging more profound shifts in features of systems that create vulnerability in the first place. In our case, these include more profound shifts in values and worldviews and resulting action on addressing the vulnerability of SGM in relation to climate change within national adaptation planning processes. Building on existing literature, references to transformation relate to transformative *capacity*, focusing on identifying efforts that have the capacity to produce transformative change according to prevailing literature and practice, rather than actual transformative outcomes^100^. Given the fledgling state of literature and practice in this field, the latter would be unfeasible to analyze, though should be taken up in future studies as practical experience accumulates in this field.

In coding the data, every thematic passage of text that mentioned the inclusion of SGM within the included NAPs was used as our coding unit. A generic coding unit like this was necessary because of the lack of uniformity in how and where SGM were sometimes discussed in the documents, which was also often brief. Because of the brevity of the text analyzed, the use of specific qualitative data analysis software was not deemed necessary. Rather, collaborative coding was done in a spreadsheet tool (Google Sheets). While the coding unit was the individual passages of text, our unit of analysis was the individual country NAPs (or a shared reading of the supporting documentation in their absence), as this was the focus of our study. Information collected about these processes sometimes came from multiple documents when used (e.g., NDC and AC documentation together).

### Data synthesis

The data were mostly presented in this study in terms of descriptive statistics, i.e., differences in distribution of how different countries adaptation planning documentation was coded. To be able to further comment on values and worldviews behind SGM inclusion in adaptation planning, we conducted further statistical analysis of differences between countries that did and did not include them on the basis of different measures of acceptance of SGM from the Equaldex, as described in the main text of this paper. This statistical analysis was done through a Wilcoxon Rank Sum test using R version 4.4.1 (2024-06-14 ucrt). This test is used to examine the association between two groups (here, countries that did and did not include SGMs in their national adaptation planning based on the systematic mapping) based on non-parametric interval data^101^. This is our case, given that the Equaldex data has equal intervals between values, though has no true 0 value in the absolute sense.

It should be noted that while Equaldex is a robust and widely applied index to understand legal and social acceptance of SGM across different countries, it must be acknowledged that it is a generalization at a high level of abstraction (i.e., national level). It is only used here to provide a general overview of the differences between the social acceptance and legal protection of SGM among the studied countries that could provide insight towards identifying levers of social change through adaptation planning.

## Supplementary information

44168_2026_363_MOESM1_ESM

44168_2026_363_MOESM2_ESM

## Data Availability

Links to all data used for the analysis done in this paper are available in Supplementary Data 1.

## Code availability

No custom code was used to generate or analyze datasets within this paper.
