# Supplementary material for: Mapping sexual and gender minority inclusion in national adaptation plans globally
Source: NPJ Clim Action. 2026 Jul 27;5(1):67. doi: 10.1038/s44168-026-00363-5 (PMC13407174; doi:10.1038/s44168-026-00363-5)
Supplement: Supplementary file 1 — 44168_2026_363_MOESM1_ESM [file 44168_2026_363_MOESM1_ESM.pdf]

Supplementary Information to "Global Mapping of the Inclusion of Sexual and Gender Minorities in National Adaptation Plans"

Supplementary Table 1. Summary of literature supporting Figure 1 in main manuscript on specific climate impacts on SGM.

**General climate impact area**	**Specific impacts**	**Disproportionate impact on SGM**
Rising temperatures	Heatwaves	Lower thermal comfort; difficulties in accessing shelters to cool down among homeless SGM, which is especially true of transgender people who are disproportionately unhoused[1].
Changes in mean precipitation	*Flooding*	Mold may grow in homes which can disproportionately impact LGBTQ+ communities due to higher rates of asthma and HIV/AIDS[2].
	Water availability (including quality)	Difficulty in accessing clean water, which is worse for those with HIV/AIDS potentially due to opportunistic infections; transgender people are much more likely to experience water insecurity compared to cisgender people; sexual and gender minorities are more likely to experience anxiety and depression due to water insecurity compared to non-sexual and gender minorities; transgender and gender diverse youth are given water demanding chores and experience disproportionate disaster impacts in the Philippines [3,4].
	Waterborne disease	Men who have sex with men with HIV are at higher odds for contracting cryptosporidiosis due to contaminated water after heavy precipitation events[5].
Coastal	*Tropical cyclones*	Discrimination and violence within temporary emergency shelters; increased food insecurity, water insecurity, and social isolation; gender-based violence; displacement (more likely to last longer or be permanent); refused access to necessary disaster and health services; difficulties in navigating disaster relief services; loss of employment; loss of culturally relevant spaces; blame from faith-based communities[6–10].
	Sea-level rise	Same-sex couples are more likely to live in areas that are adversely impacted by sea-level rise[11].
Food systems	*Food prices*	Increased food insecurity after disasters can be worsened by increasing food prices. LGBTQ+ people experience higher rates of food insecurity up to one month after a disaster. LGBTQ+ people experiencing food insecurity can experience higher risk of mental health outcomes and transactional sex[12–14].
	Malnutrition	People living with HIV/AIDS experience impacts to nutrition during wildfires such as running out of food or an increase in low nutritious foods (e.g. fast food)[15,16].

Other cross-cutting social impacts	*Social conflict*	LGBTQ+ people, especially gender non-conforming and transgender people, experience gender-based violence, such as victimization and multiple forms of violence, during and after disasters. This can impact the mental and physical health and well-being of LGBTQ+ people[17–20].
	Displacement & migration	Sexual and gender minorities are at higher risk for permanent displacement due to disasters. During migration due to climate-related events, sexual and gender minorities experience heightened discrimination and violence[12,13,21].
	Within-country economic inequality	Sexual and gender minorities experience higher rates of poverty and other economic disparities reducing capacity to prepare and adapt to climate change[6,21].

Supplementary Table 2. Description of country NAPs that include SGM.

Country	Region	Year	Summary	Document Type	Term(s) Used	Transformative Capacity
Argentina	South America	2022	Multiple references to LGBTI+ in relation to inclusion in decision-making processes and building adaptive capacity. Includes axes of sovereignty, habitability, and care for both "cis heterosexual women and LGBTI+" people. Refers to numerous specific actions and metrics to evaluate how LGBTI+ people are better included in planning processes.	National Adaptation Plan	Lesbian, gay, bisexual, transgender, transsexual, transvestite, intersex and any non-binary identity (LGBTI+)	Transformative (Level 3)
Canada	North America	2023	Recognition of the increased severity of impacts on 2SLGBTI+ and their lack of procedural and geographical (distributional) inclusion in social determinants of health that are impacted by	National Adaptation Strategy; National Adaptation Action Plan	Two-spirit, lesbian, bisexual, transgender, queer, intersex, and other non-binary identities (2SLGBTQI+)	Sensitive (Level 1)

			climate change.			
India	Southeast Asia	2023	Mentions involvement of transgender people in Self-Help Groups (SHGs), which allows for people to pool together their resources to build financial security.	Third National Communication and Initial Adaptation Communication	Transgender	Responsive (Level 2)
New Zealand	Oceania	2022	Discusses the vulnerability of different communities to the impacts of climate change, including "rainbow and LGBTQI+ communities".	National Adaptation Plan	Rainbow; lesbian, gay, bisexual, transgender, queer, intersex, and other non-binary identities (LGBTQI+)	Sensitive (Level 1)
Uruguay	South America	2021	Discusses the inequality of distribution and recognition of climate impacts on different groups, such as trans people.	National Adaptation Plan	Trans people	Sensitive (Level 1)
Norway	Northern Europe	2022	Discusses the participation of Norway in international processes for raising awareness of LGBTI people in the context of climate change adaptation.	National Adaptation Strategy	LGBTI	Sensitive (Level 1)
Australia	Oceania	2021	Mentions the humanitarian support provided by Australia for international initiatives for changing perceptions of LGBTI people that are increasingly vulnerable to climate impacts.	Second Adaptation Communication	LGBTI	Responsive (Level 2)
Cabo Verde	Western	2022	Quotes the NAP	National	Sexual	Sensitive (Level 1)

	Africa		planning procedures that recognises, among other things, the centrality of "equality of rights, opportunities, and challenges" between various marginalised groups, including on the basis of "sexual orientation".	Adaptation Plan	orientation	
Ghana	Western Africa	2022	Mentions LGBTQI in relation to developing capacity in the collection, management, updating, sharing and use of climate-risk data. In addition, they suggest creating disaggregated data based on LGBTQI identity.	Infrastructure Resilience Plan	LGBTQI	Responsive (Level 2)
Kiribati	Oceania	2020	Discusses climate change vulnerability from an intersectional perspective, that includes sexual orientation.	National Adaptation Plan	Sexual orientation	Sensitive (Level 1)
Montenegro	Southern Europe	2023	In defining safeguards for social and environmental risks, includes the prevention of unequal or discriminatory impacts on people on the basis of gender (including transgender people) and sexual orientation.	Readiness Proposal	Sexual orientation; transgender	Sensitive (Level 1)
Vanuatu	Oceania	2021	Describes that LGBTQIA+	First Nationally Determined	LGBTQIA+	Sensitive (Level 1)

			individuals experience social stigma that place them at higher risk for climate impacts.	Contribution		
Costa Rica	Central America	2022	Includes transsexuals in decision-making processes regarding adaptation to climate change	National Adaptation Plan	Transsexual	Responsive (Level 2)
Bangladesh	Southern Asia	2023	Bangladesh includes people with gender diverse identities as part of their communities that are vulnerable. They include a robust amount of actions and indicators that they want to include gender diverse individuals in including losses and damages/climate financing, community-based adaptation, agriculture, and capacity-building and training to use climate-smart tools and techniques. They also describe ways that they can reach out to these communities to include them within the planning, implementation, and evaluation processes. Unfortunately, they do not include those who have differing sexual orientations as part of their list of	National Adaptation Plan	Diverse gender identities	Transformative (Level 3)

			vulnerable communities.			
Fiji	Oceania	2018	The Fijian government reached out for input from the Pacific Sexual and Gender Diversity Network for the NAP. They include LGBTQ communities as part of their definition of disadvantaged group. They describe that there should be an understanding of the impacts of climate change on disadvantaged groups such as LGBTQ communities and that they should be considered "active agents of change" instead of "vulnerable groups". They want to develop planning processes that empower and support disadvantaged groups to have equitable access to leadership positions, decision making processes, opportunities and resources.	National Adaptation Plan	LGBTQ	Sensitive (Level 1)
Lebanon	Western Asia	2023	Discusses that climate change is experienced differently based on existing inequalities including on the basis of sexual orientation and	Good Practices for Developing Lebanon’s National Adaptation Plan	Sexual orientation; gender identities	Sensitive (Level 1)

			gender. They also mention that the NAP processes should tackle inequalities that are worsened by climate change to deliver equitable adaptation outcomes for people of all gender identities.			
Pakistan	Southern Asia	2023	Transgender individuals were mentioned as being part of the most marginalized groups in Pakistan and that they will implement special interventions for the empowerment of vulnerable groups including transgender individuals. They also included sexual orientation in terms of supporting capacity development in regards to climate change.	National Adaptation Plan	Sexual orientation; transgender	Responsive (Level 2)
Panama	Central America	2022	Used the term LGBT in relation to outreach and communication strategies of the NAP. The purpose of the outreach and communication strategies is to develop and implement understanding and to engage stakeholders on climate change adaptation planning processes. They will communicate	Readiness Proposal	LGBT	Sensitive (Level 1)

			findings related to gender and adaptation and will conduct annual surveys to evaluate effectiveness of the strategy. This includes collecting sex-disaggregated data, but unsure if this will include cisgender, transgender, intersex, and non-binary identities.			
Peru	South America	2021	The NAP recognizes individuals can be discriminated against on the basis of sexual orientation and gender identity.	National Adaptation Plan	Sexual orientation; gender identity	Sensitive (Level 1)
Philippines	Southeast Asia	2024	When describing a starting point in fostering a gender-responsive approach within their NAP, they say that they need to recognize the diverse needs and vulnerabilities of women, girls, and gender minorities. There are many places where a gender-responsive approach is mentioned throughout their NAP related to stakeholder engagement, incentivizing participation, and identifying gaps. It is unclear if there is a clear effort to include gender minorities within	National Adaptation Plan	Gender minorities	Sensitive (Level 1)

			this.			
Saint Vincent and the Grenadines	Caribbean	2019	Mentions "sexuality" once in regards to a Food and Agriculture Organization (FAO) definition on what a gender analysis should entail.	National Adaptation Plan	Sexuality	Sensitive (Level 1)
United States	North America	2024	There is no overall national adaptation plan for the US, rather there are agency specific national adaptation plans. Out of 28 agency plans, the Department of Health and Human Services is the only agency whose plans mention sexual and gender minorities. This plan states that these groups have experienced historic discrimination and whose health is disproportionately impacted by climate change.<br><br>*Based on recent federal changes, it is possible that this adaptation plan will not be available in the future.	Health and Human Services Climate Action Plan	Sexual and gender minorities	Sensitive (Level 1)
Mexico	North America	2022 and 2025	The First Nationally Determined Contribution mentions that climate action should include and promote LGBTQ rights.<br>The First Biennial Transparency Report integrates	First Nationally Determined Contribution and First Biennial Transparency Report	LGBTQ, LGBTIQ+, LGBTTTIQ+	Responsive (Level 2)

			the needs of sexual and gender minorities by promoting inclusive policies that address their unique vulnerabilities to climate change. It emphasizes the participation of LGBTIQ+ people in national technological and community energy projects, while also considering gender identity and sexual orientation in the design of climate policies. Furthermore, it advocates for inclusive communication strategies and the strengthening of public and social institutions' capacity to implement ecosystem adaptation measures with a gender and human rights perspective.			

Supplementary Table 3. Full p value, rg (Glass rank biserial correlation coefficient effect size for Mann-Whitney two-sample rank-sum test) including lower and upper confidence intervals (ci).

Metric	p	rg	Lower ci	Upper ci
Legal	0.0141	-0.331	-0.572	-0.0666
Public opinion	0.0014	-0.430	-0.661	-0.1790

References

1. Mazzone, A. Thermal comfort and gender affirmation: A virtual ethnography of extreme heat among trans women in Rio de Janeiro. *Soc. Sci. Med.* **362**, 117481 (2024).
2. Chow, N. A. *et al.* Hurricane-Associated Mold Exposures Among Patients at Risk for Invasive Mold Infections After Hurricane Harvey — Houston, Texas, 2017. *MMWR Morb. Mortal. Wkly. Rep.* **68**, 469–473 (2019).
3. Brewis, A. *et al.* Gender identities, water insecurity, and risk: Re-theorizing the connections for a gender-inclusive toolkit for water insecurity research. *WIREs Water* **11**, e1685 (2024).
4. Logie, C. H. *et al.* Associations between water insecurity and mental health outcomes among lesbian, gay, bisexual, transgender and queer persons in Bangkok, Thailand and Mumbai, India: Cross-sectional survey findings. *Camb. Prisms Glob. Ment. Health* **11**, e31 (2024).
5. Inungu, J. N., Morse, A. A. & Gordon, C. Risk factors, seasonality, and trends of cryptosporidiosis among patients infected with human immunodeficiency virus. *Am. J. Trop. Med. Hyg.* **62**, 384–387 (2000).
6. Goldsmith, L., Raditz, V. & Méndez, M. Queer and present danger: understanding the disparate impacts of disasters on LGBTQ+ communities. *Disasters* **46**, 946–973 (2022).
7. Gaillard, J. C. *et al.* Beyond men and women: a critical perspective on gender and disaster. *Disasters* **41**, 429–447 (2017).
8. Gaillard, J. C., Gorman-Murray, A. & Fordham, M. Sexual and gender minorities in disaster. *Gend. Place Cult.* **24**, 18–26 (2017).
9. Gorman-Murray, A., McKinnon, S. & Dominey-Howes, D. Queer domicide: LGBT displacement and home loss in natural disaster impact, response, and recovery. *Home Cult.* **11**, 237–261 (2014).

10. Gorman-Murray, A., McKinnon, S., Dominey-Howes, D., Nash, C. J. & Bolton, R. Listening and learning: Giving voice to trans experiences of disasters. *Gend. Place Cult.* **25**, 166–187 (2018).
11. Mahowald, L. & Shaw, A. *Climate Change Risk for LGBT People in the US*. (UCLA Williams Institute, 2024).
12. Aung, T. W. & Sehgal, A. R. Prevalence, Correlates, and Impacts of Displacement Because of Natural Disasters in the United States From 2022 to 2023. *Am. J. Public Health* **115**, 55–65 (2025).
13. Geiger, J., Mendez, M. & Goldsmith, L. *Amplified Harm: LGBTQ+ Disaster Displacement*. (University of California, Irvine, 2023).
14. Murphy, N. *et al.* Changing climates, compounding challenges: a participatory study on how disasters affect the sexual and reproductive health and rights of young people in Fiji. *BMJ Glob. Health* **8**, e013299 (2023).
15. Saberi, P., Ming, K., Arnold, E. A., Leddy, A. M. & Weiser, S. D. Extreme weather events and HIV: Development of a conceptual framework through qualitative interviews with people with HIV impacted by the California wildfires and their clinicians. *BMC Public Health* **23**, 950 (2023).
16. Native Women’s Association of Canada Environmental. *TOOLKIT – IMPACT OF CLIMATE CHANGE on Indigenous Women, Girls, Two-Spirit, Transgender and Gender-Diverse People*. https://nwac.ca/assets-documents/EIPCCP-Climate-Change-and-Biodiversity-Toolkit-2023-2024-FINAL.pdf (2024).
17. Van Daalen, K. R. *et al.* Extreme events and gender-based violence: a mixed-methods systematic review. *Lancet Planet. Health* **6**, e504–e523 (2022).
18. Norwegian Red Cross. *“That Never Happens Here”: Sexual and Gender-Based Violence against Men, Boys and/Including LGBTQ+ Persons in Humanitarian Settings*. https://www.icrc.org/sites/default/files/document_new/file_list/that_never_happens_here_report_-_sexual_violence.pdf (2022).

19. Parzniewski, S. *et al.* Factors affecting the risk of gender-based violence among 2SLGBTQIA+ adolescents and youth: a scoping review of climate change-related vulnerabilities. *Front. Sociol.* **10**, 1541039 (2025).
20. Outright International. *LGBTQ LIVES IN CONFLICT AND CRISIS A Queer Agenda for Peace, Security, and Accountability*. (2023).
21. Mann, S., McKay, T. & Gonzales, G. Climate Change-Related Disasters & the Health of LGBTQ+ Populations. *J. Clim. Change Health* **100304**, (2024).
